# Methods for sampling geographically mobile female traders in an East African market setting

**DOI:** 10.1371/journal.pone.0190395

**Published:** 2018-01-11

**Authors:** Aimee Leidich, Lillian Achiro, Zachary A. Kwena, Willi McFarland, Torsten B. Neilands, Craig R. Cohen, Elizabeth A. Bukusi, Carol S. Camlin

**Affiliations:** 1 mSurvey, San Francisco, CA, United States of America; 2 Centre for Microbiology Research, Kenya Medical Research Institute (KEMRI), Nairobi, Kenya; 3 Global Health Sciences, University of California at San Francisco (UCSF), San Francisco, CA, United States of America; 4 Center for AIDS Prevention Studies, University of California at San Francisco (UCSF), San Francisco, CA, United States of America; 5 Department of Obstetrics, Gynecology & Reproductive Sciences, University of California, San Francisco (UCSF), San Francisco, CA, United States of America; Public Library of Science, UNITED KINGDOM

## Abstract

**Background:**

The role of migration in the spread of HIV in sub-Saharan Africa is well-documented. Yet migration and HIV research have often focused on HIV risks to male migrants and their partners, or migrants overall, often failing to measure the risks to women via their direct involvement in migration. Inconsistent measures of mobility, gender biases in those measures, and limited data sources for sex-specific population-based estimates of mobility have contributed to a paucity of research on the HIV prevention and care needs of migrant and highly mobile women. This study addresses an urgent need for novel methods for developing probability-based, systematic samples of highly mobile women, focusing on a population of female traders operating out of one of the largest open air markets in East Africa. Our method involves three stages: 1.) identification and mapping of all market stall locations using Global Positioning System (GPS) coordinates; 2.) using female market vendor stall GPS coordinates to build the sampling frame using replicates; and 3.) using maps and GPS data for recruitment of study participants.

**Results:**

The location of 6,390 vendor stalls were mapped using GPS. Of these, 4,064 stalls occupied by women (63.6%) were used to draw four replicates of 128 stalls each, and a fifth replicate of 15 pre-selected random alternates for a total of 527 stalls assigned to one of five replicates. Staff visited 323 stalls from the first three replicates and from these successfully recruited 306 female vendors into the study for a participation rate of 94.7%. Mobilization strategies and involving traders association representatives in participant recruitment were critical to the study’s success.

**Conclusion:**

The study’s high participation rate suggests that this geospatial sampling method holds promise for development of probability-based samples in other settings that serve as transport hubs for highly mobile populations.

## Introduction

Population movements over the last century have determined the speed, direction and burden of infectious disease within places. In the case of HIV/AIDS, ample historic evidence shows that the transfer of people between urban areas and from urban to rural areas provided the vehicle for the spread of the epidemic [1–5]. In sub-Saharan Africa, informal settlements or peri-urban areas where migrants often take up temporary residence have been shown to have rates of HIV twice as high as urban and rural areas [5–7] making migrants one of the groups most vulnerable to HIV acquisition according to the Joint United Nations Program on HIV [8]. Research to help us understand this highly vulnerable group exists but is limited by methodological barriers to developing population-based sampling frames for populations in motion [9].

This is particularly true for research on women’s migration in sub-Saharan Africa. Studies have documented a feminization of internal migration across the region in recent decades [6, 10–14] showing that women are migrating internally at an equal and in some cases higher rate than men [15, 16]. Furthermore, studies have documented high HIV acquisition and transmission risks among female migrants and higher HIV prevalence and risk behavior among migrant compared to non-migrant women [1, 6, 17–21] and even to migrant men [1, 22]. Still, studies of migration and HIV in sub-Saharan Africa have largely focused on male migrants and their partners [3, 23–26], only studying sex-specific migration patterns, only (or largely) interviewing male heads of households, identifying migrants in terms of large administrative units (e.g. states), and using measures that under-represent women’s labor force participation and migration [1, 27].

To improve methods for quantifying and characterizing female migrants and their HIV risks, this study describes a novel method for sampling a group of highly mobile women in Kisumu Kenya: female market vendors selling goods at Kibuye Market. Kibuye Market is reputed to be one of the largest outdoor markets in Eastern Africa, attracting predominantly female vendors from across Uganda, Tanzania, and Kenya to take up temporary residence and sell goods to support their families. Beyond being a shared place where female migrants congregate to sell their goods, Kibuye Market is located in Kisumu municipality which has an HIV prevalence of 15.1% among women aged 15–49 [28, 29] compared to a national prevalence of 6.9% among women aged 15–64 [29, 30]. This market provides a temporary space to sample migrant women at high risk of HIV transmission who would otherwise be difficult to identify and reach given their high levels of mobility.

In an analysis of the survey data collected in this study, published recently [31], our team estimated HIV prevalence and fitted logistic regression models to measure associations between mobility, risk behaviors, and HIV infection among the female market traders. Key findings were that, as expected, levels of both mobility and HIV prevalence were high in this population, compared with the general population of women of reproductive age in Kisumu, in Kenya and the region: HIV prevalence among the female market traders was 25.6% (95% CI 21.0–30.8); 11.5% had migrated (changed residence over county or national boundary) in the past year, and 39.3% had migrated in the past five years. Over one-third (38.3%) spent nights away from their main residence in the past month, with 11.4% spending more than a week away. Multiple partners in the last year were reported by 13.1% of women, and 16% of married women reported a concurrent partnership. Our explanations for these findings were informed by our prior qualitative work in the population [32], which described the contextual and behavioral risks faced by migrant and highly mobile women in the region, including market traders.

This paper provides a detailed description of the innovative sampling method that we developed and tested in order to estimate mobility and HIV prevalence among female market traders. We describe a method for using Geographical Information System (GIS) data to record the market stall location of every stall in a large open air market in East Africa, Kenya, and using these locations to create a random sample of stalls occupied by female market vendors. This paper reviews our sampling techniques, lessons learned from this method, and selected characteristics of a highly mobile population of women in Kenya. These methods and lessons learned may have broad applicability for similar efforts in other mobile populations in sub-Saharan Africa.

## Methods

This study is part of a larger research program investigating migration, mobility and HIV risk among women in the Nyanza region of Kenya. Previously published studies aimed to characterize the contexts and processes that may facilitate HIV acquisition and transmission among migrant and highly mobile women in the Kisumu area of Nyanza Province, Kenya [32, 33]. This research program sought to measure mobility patterns and HIV prevalence among women who were **migrant** (defined as having undertaken a permanent change of residence as an adult, not for purposes of marriage, over a district, provincial, or national boundary) or **highly mobile** (defined as having traveled away from the area of primary residence for the purpose of business or livelihood, involving sleeping away from home at least once per month, in the past year). Our team’s prior qualitative research identified typologies of female migrants and highly mobile women in the Kisumu area of western Kenya which included female market traders [34]. The present study, a component of this larger research program, sought to measure migration, mobility and HIV prevalence among female market traders who work at the largest open air market in Kisumu (Kibuye). These traders, some of whom we knew to be highly mobile, and some of whom we knew to be residentially stable, are engaged in exclusively informal sector work, in small-scale trade commonly of clothing and shoes, cosmetics, fish, grains/cereals and vegetables. This article describes the rigorous, innovative approach we used to obtain a probability-based sample of the entire market for a subsequent survey of mobility and HIV prevalence among female traders. The sampling occurred in three stages: 1.) identification and mapping of all market stall locations using Global Positioning System (GPS) coordinates; 2.) using female market vendor stall GPS coordinates to build the sampling frame using replicates; and 3.) using maps and GPS data for recruitment of study participants.

### Stage 1: Identification and mapping of all market stall locations using GPS coordinates

Following a period of mobilization and site preparation, every stall or space selling goods at the Sunday Kibuye Market was visited by study staff. The Sunday market was chosen for data collection in order to include additional vendors who only travel to Kibuye Market for the larger Sunday market. During these Sunday visits tablets equipped with GPS were used to collect data on the geographic location of each vendor (i.e., the GPS coordinate); the gender of the vendor; the stall type (e.g., a temporary stall, an open-air space on the ground, or a permanent, roofed stall); the type of wares being sold; and any other identifiers or comments to help locate the stall upon return such as nearby landmarks or a visual description of the stall. Each stall was assigned a unique identifier at that time.

### Stage 2: Using female market vendor stall GPS coordinates to build the sampling frame using replicates

An initial sample size for this study was derived using the following formula based on the normal approximation to the binomial distribution: *n* = *z2 p*(1-*p*)/*d2* where *p* is HIV prevalence (*p* = 25%), *d* is the precision with which the prevalence will reflect the true value (*d* = 5% of the true value or with 95% confidence) and *z* is the critical value of the confidence interval for a standard normal distribution (*z* = 1.96), equaling 289 [35]. A more exact method was then used to compute the final sample size of 306 using the exact Clopper-Pearson two-sided confidence intervals for proportions generated with NCSS PASS software [36]. Sample size was calculated based on the assumption of a 25% or higher HIV prevalence among female traders at Kibuye market, given prior studies in the region showing higher HIV prevalence among female migrants [1, 17, 18, 37–41], and women in Kisumu [42]. Because the exact method makes fewer assumptions than the normal approximation method, it is more conservative and thus the study recruited 306 participants.

To ensure meeting the sample size target of 306, the sample was drawn in "replicates." In this approach, each replicate is a systematic random sample of the whole market place gauged to be a fraction of the whole sample size. The survey is done by undertaking one replicate at a time, completing the replicate, and moving to the next until the minimum number of replicates completes the whole sample.

These replicates eased logistical planning and allowed for scaling up or down the sample size in the field while still maintaining theoretical rigor. If the projection initially undershot the target (e.g., by participation, eligibility, availability, or turn-over), then additional replicates could be added without violating the equal probability of any woman in the market being included and therefore the representation of market women as a whole. Similarly, if the projection initially overshot the target, then only the current replicate need be completed and the sampling integrity would be maintained.

Assuming 80% of women from each replicate would participate in the study, the number of female vendor stalls assigned to each replicate was increased to 128 for a total of 384 possible contacts across three replicates. In case the contact rate was 75% or lower across the first three replicates, a fourth replicate of 128 randomly assigned stalls was added. Lastly, a fifth replicate was added with 15 pre-selected random alternates to supplement five alternatives for each of the three initial replicates. If less than 80% of vendors were recruited from any of the previous replicates, staff would select from this fifth replicate so the study would not recruit any more than 5% random alternates. This added up to a total of 527 stalls occupied by female vendors being assigned to one of five replicates.

The location and unique ID of all female vendors by replicate were mapped separately and overlaid on a remote sensing image of Kibuye Market. Each replicate map was magnified by market quadrant (i.e., NE, NW, SE, SW) to aid in locating stalls that were close to each other on a single replicate map. Study staff completed each replicate sequentially until the target sample was reached.

### Stage 3: Using maps and GPS data for recruitment of study participants

Using the replicate maps as described above, teams of six selected a vendor ID on the map to visit, cross referenced the vendor ID with GPS coordinate data for that ID and then used the GPS devices on the tablets to direct them to the vendor location. If the prospective stall was still occupied by a female vendor, she would be given a verbal description of the study and requested to walk with study staff to the study site while a neighbor, family member, friend, or study staff member watched over her stall. Female vendors who were not comfortable leaving their stall would be given instruction on how to visit the study site on their own and would be followed up with either in person or using the study phone until they appeared at the study site. If a prospective stall was vacant or occupied by a man, neighboring vendors would be asked to share the whereabouts of the female vendor who occupied the stall previously and recommendations on how to reach her. If neighboring vendors were unsure of her whereabouts, staff would wait for a minimum of 45 minutes for the vendor’s return and would revisit that stall at least one time or more that day, as convenient, and for three subsequent Sundays for a total of five attempts to make contact with the original female vendor. Any female vendors not identified at this time would be considered “non-contact.”

Once a study participant arrived to the study site (on weekdays designated as a local Health Center and on weekends designated as a nearby primary school) she would provide written, informed consent to participate in a short survey interview and have an opportunity to ask questions. During the interview, she would be asked about her basic demographics, mobility and migration patterns. After interviewing, she would move to a second room where she would provide a separate written informed consent for HIV Voluntary Counseling and Testing. It was not required that those who participated in the survey also had to participate in testing. Those who consented were tested with the Alere Determine™ HIV 1/2 test (Waltham, MA, USA) and provided results on the spot. Anyone found to be HIV positive would be given a confirmatory rapid test (SD Bioline, Yongin-si, Gyeonggi-do, Republic of Korea) during the same session. If results from the Determine and Bioline tests conflicted, then Unigold® (Bray, Co Wicklow, Ireland) was used as a tie breaker. If results were confirmed to be HIV-positive, participants were counseled and referred for HIV care and treatment at one of the Family AIDS Care & Education Services (FACES)-supported clinics in Kisumu [34]. The entire process from arrival to the study site, consent, interview and testing generally took less than one hour with the most time being spent on the consent and counseling (estimated to be 15 minutes per participant). Participants received a total of Ksh. 500 ($6.20) in exchange for the opportunity costs associated with leaving their market stalls on busy trading days. The study protocol was approved by the KEMRI Ethical Review Committee (#2361) and the Committee on Human Research at the University of California at San Francisco (#10–02936).

## Results

### Mapping of market stalls

**Fig 1:**
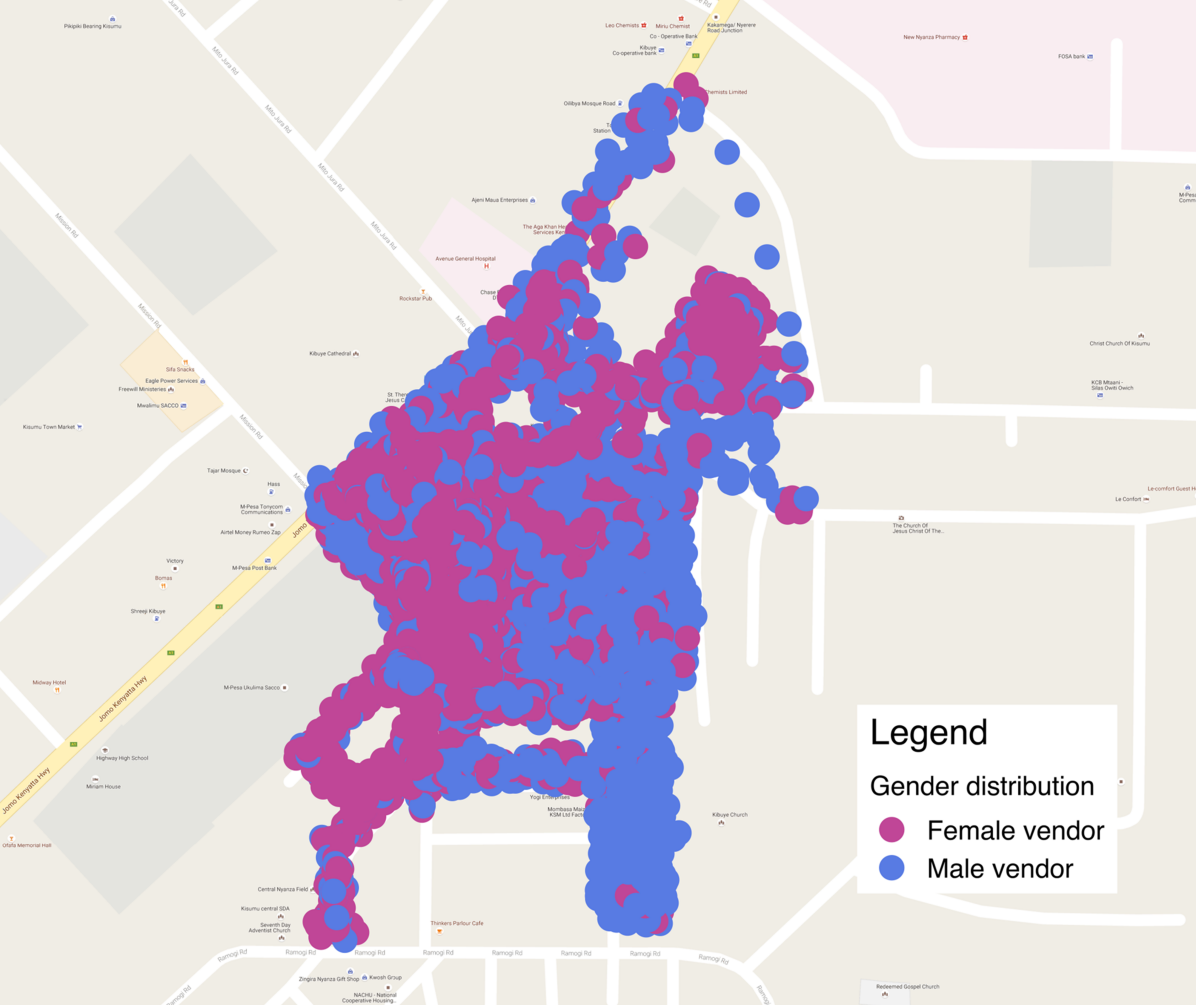
Vendor locations* by gender, Kibuye Market, Kisumu, Kenya, 2013. *Specific vendor locations have been blurred and in some cases removed to protect participant privacy.

Stage one, occurred from September to December, 2013. During this time, the GPS locations of 6,390 vendor stalls were gathered, of which more than two-thirds (63.6%, n = 4,064/6,390) were occupied by women. Most stalls in Kibuye were located in the NE quadrant (35.7%, n = 2,280/6,390) and the fewest in the SE quadrant (17.0%, n = 1,088/6,390) which was also occupied predominantly by male vendors (62.2%, n = 677/1,088; Table 1 and Fig 1). Over half (55.8%, n = 3,568/6,390) of the structures in Kibuye Market were considered temporary or a space on the ground and these were predominantly occupied by women (75.6%, n = 2,699/3,568). The main known wares sold were food (23.4%, n = 1,497/6,390) and clothing, shoes, and accessories (22.3%, n = 1,424/6,390) all of which were mostly sold by women (Table 1).

**Table 1:** Description of Kibuye Market vendors by gender, Kisumu, Kenya, 2013.

	Female (n = 4,064)		Male (2,326)	
	%	n	%	n
**Type of stall**				
Permanent (roofed)	48.4%	1365	51.6%	1457
Temporary (ground)	75.6%	2699	24.4%	869
**Type of wares**				
Beauty products / cosmetics	83.6%	51	16.4%	10
Cloth/clothing/shoes/accessories	71.7%	1021	28.3%	403
Food	89.9%	1346	10.1%	151
Food kiosk	81.5%	22	18.5%	5
Furniture	6.4%	11	93.6%	162
Hardware	5.8%	18	94.2%	290
Herbs and medicines	86.8%	33	13.2%	5
Hotel	73.9%	65	26.1%	23
Housewares / cleaning products	73.2%	104	26.8%	38
Utensils / cookware	50.0%	44	50.0%	44
Other	45.1%	157	54.9%	191
Unknown	54.3%	1192	45.7%	1004
**Quadrant**	** **	** **	** **	** **
NE	61.9%	1411	38.1%	869
NW	69.6%	1252	30.4%	547
SE	37.8%	411	62.2%	677
SW	80.9%	990	19.1%	233

### Building the sampling frame and recruitment of individuals

Stage two occurred immediately after Stage 1 from January to June 2014. Out of the five replicates drawn, our sample size was reached after visiting 323 stalls from the first three replicates. Of these, 306 female vendors were identified and successfully recruited into the study for a participation rate of 94.7%. Among the 17 vendors approached but not recruited into the study, 7 refused participation [husband didn’t approve of participation (n = 1), vendor was a minor and parents didn’t approve of participation (n = 1), female vendor refused participation (n = 5)] and 10 were considered non-contact [stall vacant after five visits (n = 4), stall newly occupied by a male (n = 6)]. Data were inadvertently lost for 1 participant; hence primary study findings presented here and elsewhere [31] are for n = 305 participants.

### Lessons learned recruiting study participants

Recruiting participants after mapping their GPS coordinates was the most time-consuming part of the study, sometimes taking teams over 20 minutes to find a single vendor. In instances where more time was needed to locate a vendor, the reasons for needing more time included movement of vendors from one location during weekdays to a different location during the larger Sunday market; vendors holding more than one stall or use of a stall and storage unit that they only occasionally frequent; the construction of new stalls over the study period; and difficulty deciphering the vendor ID on the replicate maps. To rectify these challenges, study staff frequently communicated with stall neighbors about absent or different vendors and whether they had recently moved away or died and leads as to where they could be found. Replicate maps were also “magnified” into four separate maps showing stalls in the NE, NW, SE, and SW quadrants of the market to help study staff better see each vendor’s location and Unique ID.

The number of contacts needed to find a woman varied, depending on whether a woman used her stall only on Sundays or also during the week. Most women were contacted within three attempts, and only in rare instances were five full attempts made. The average time between first contact and enrollment was about seven days, especially for those who were only contacted on the Sunday market day. The number of reminders needed to ensure appointments were attended also differed greatly between those who had someone to tend to their stall, versus those who did not; on average about five reminders were needed. During the data collection period, the team met some of the women every day making it easy to check in with them in person or via cell phone. In summary, the study was labor-intensive, yet feasible to be conducted with a small research grant of $40,000 in direct costs (to pay mostly for personnel and equipment in the field site). This was supplemented by an additional amount of $6,400 in funds to extend the study data collection period for two months, due to disruptions to the data collection schedule because of civil unrest in the study site (disputes between market traders and the municipal authority, which were resolved by the end of the study).

In this context of civil unrest, some vendors mistrusted the study staff, which made recruitment challenging and sometimes dangerous for staff. This mistrust was attributed to several factors such as a tense relationship between market vendors and the Kisumu Municipal Council who some vendors assumed partnered with the study in order to confiscate their land; a general misunderstanding of what research was and why it is valuable; fear that talking to others, especially of the opposite sex, would provoke harm from husbands. To encourage cooperation and trust, staff conducted a round table meeting with the traders union and section heads at Lumumba Health Center to dispel any myths about the study. Female staff were used to accompany female vendors to the study site, and in certain cases seek prior permission from the husband. As more vendors participated, neighbors encouraged others to participate reassuring them the survey was legitimate and short.

## Discussion

Understanding the people that occupy a certain place, whether permanently or temporarily, is the foundation for understanding population health. The health of migrant populations is especially challenging to understand since by definition migratory groups continuously move from one point to the next making their ‘place’ hard to identify [43]. This paper describes a novel method for sampling a highly mobile female population of women who work in the informal labor sector as market vendors at a large East African market. To our knowledge, this is the first study of its kind to create a truly representative sample of female market vendors by mapping the location of every market stall and then using these locations to set the sampling frame.

Identifying the appropriate spatial scale is a major methodological challenge when trying to measure HIV-related behaviors among mobile populations [43]. This is especially true of female market vendors given the lack of formal censuses of this population, the shifting composition of the population, and the complexities of patterns of mobility among female traders. Sampling this population provided rich information about the characteristics of different vendors and recruitment and interpersonal challenges faced by researchers and staff working in a market setting.

The methodological innovation of this study involved geospatial mapping of market stalls by gender on the main, weekly market day to optimize construction of a sampling frame. We learned that despite their high mobility, market traders are socially interconnected; while it was not unusual to find an empty stall that was usually used by a given trader, other traders tended to know whether the occupant had ‘quit business’ or not. Those who used market stalls occasionally or seasonally would usually find someone to use the space in their absence. Thus, we feel that this methodology is broadly applicable for data collection in other highly mobile populations who circulate through given spaces, and form social relationships with others in those spaces.

Mapping vendor locations showed that market vendors at Kibuye Market were predominantly female. Despite holding the majority, females disproportionately occupied temporary or ground spaces for selling their goods. Males more often occupied the longer-lasting and more lucrative selling locations, having to our knowledge, on the basis of preliminary research, more initial capital to rent larger and more permanent spaces and to purchase larger stocks of goods. Similarly, we observed that the sale of goods was divided along gender lines with furniture and hardware vendors being almost entirely male.

A limitation of this study is that a gold standard does not exist for validating this sampling methodology. While researchers have theorized innovative measurement and documentation of the movement of people, there are few data for the calculation of unbiased population metrics to compare statistically significant differences between migratory individuals and groups [44]. A recent study among highly mobile fisher folk in fishing communities used a similar sampling approach by creating replicates from a global list of fisher folk registered at each fish-landing beach and using the replicates to systematically replace confirmed non-contacts or refusals until the study sample size was reached [45]. Still, additional research replicating this method is needed to better understand the value of setting a sampling frame based on the mapped location of mobile individuals over a given time period.

Notwithstanding this limitation, mapping each vendor location permitted the development and recruitment of a probability-based sample of an under-researched, and hard to reach population of migrant and highly mobile women—a population that has been recognized to be at high risk of HIV acquisition and transmission, but whose mobility is not well-characterized or understood [1, 6, 17–21].

## Conclusion

The study’s high participation rate suggests that this geospatial sampling method holds promise for development of probability-based samples in other settings that serve as transport hubs for highly mobile populations. This is attributable to efforts undertaken to build trust with market stakeholders in order to gain entry to the market and positive feedback from female vendors who participated in the survey. The study team’s experience suggests that the extra time and effort taken to prepare this setting for research activities yielded value for informing efforts to obtain probability-based samples of highly mobile populations.

## References

[1] Camlin CS, Hosegood V, Newell ML, McGrath N, Barnighausen T, Snow RC. Gender, migration and HIV in rural KwaZulu-Natal, South Africa. PloS one. 2010;5(7):e11539 Epub 2010/07/17. doi: 10.1371/journal.pone.0011539 ; PubMed Central PMCID: PMCPMC2902532. 20634965 10.1371/journal.pone.0011539 PMC2902532

[2] Glynn JR, Ponnighaus J, Crampin AC, Sibande F, Sichali L, Nkhosa P, et al The development of the HIV epidemic in Karonga District, Malawi. AIDS (London, England). 2001;15(15):2025–9. Epub 2001/10/16. . 11600832 10.1097/00002030-200110190-00016

[3] Jochelson K, Mothibeli M, Leger JP. Human immunodeficiency virus and migrant labor in South Africa. International journal of health services: planning, administration, evaluation. 1991;21(1):157–73. Epub 1991/01/01. doi: 10.2190/11UE-L88J-46HN-HR0K . 2004869 10.2190/11UE-L88J-46HN-HR0K

[4] Garin B, Jeannel D, Kazadi K, Combe P, Singa L, De The G. Introduction of HIV-1 in a rural city of Zaire. Annales de la Societe belge de medecine tropicale. 1993;73(2):143–7. Epub 1993/06/01. . 8368890

[5] Coffee MP, Garnett GP, Mlilo M, Voeten HA, Chandiwana S, Gregson S. Patterns of movement and risk of HIV infection in rural Zimbabwe. The Journal of infectious diseases. 2005;191 Suppl 1:S159–67. Epub 2005/01/01. doi: 10.1086/425270 . 15627226 10.1086/425270

[6] Boerma JT, Urassa M, Nnko S, Ng'weshemi J, Isingo R, Zaba B, et al Sociodemographic context of the AIDS epidemic in a rural area in Tanzania with a focus on people's mobility and marriage. Sexually transmitted infections. 2002;78 Suppl 1:i97–105. Epub 2002/06/27. doi: 10.1136/sti.78.suppl_1.i97 ; PubMed Central PMCID: PMCPMC1765836. 12083453 10.1136/sti.78.suppl_1.i97 PMC1765836

[7] Shisana O, Rehle T, Simbayi LC, Parker W, Zuma K, Bhana A, et al. South African national HIV prevalence, HIV incidence, behavior and communication survey, 2005. Cape Town, South Africa: 2005.

[8] HIV/AIDS JUNPo. The Gap Report 2014: Migrants. Geneva: 2014.

[9] Kearns RaM, G. From medical to health geography: novelty, place and theory after a decade of change. Progress in Human Geography. 2002;26(5).

[10] Collinson M, Tollman SM, Kahn K, Clark SJ, and Garenne M. Highly prevalent circular migration: households, mobility and economic status in rural South Africa. Africa on the move: African migration and urbanisation in comparative perspective: Wits University Press; 2006.

[11] McDonald D. On Borders: Perspectives on International Migration in Southern Africa Dodson B, editor. Kingston, Ontario, Canada/New York: Southern African Migration Project/St. Martin's Press; 2000.

[12] Hugo GJ. Internal migration of women in developing countries. Proceedings of the United Nations Expert Meeting on the Feminization of Internal Migration, Aguascalientes, Mexico, 22–25 October 1991. New York, New York: United Nations, 1993.

[13] Posel D. Have migration patterns in post-apartheid South Africa changed? Journal of Interdisciplinary Economics. 2004;15:277–92.

[14] Tienda M. Africa on the move: African migration and urbanisation in comparative perspective Zlotnik H, editor. Johannesburg: Witerstrand University Press; 2006.

[15] Bilsborrow RE. Preliminary report of the United Nations Expert Group Meeting on the feminization of internal migration. International Migration Review. 1992;26:138–61.

[16] Zlotnik H. The global dimensions of female migration 2003 [cited 2015 October 17]. Available from: http://www.migrationpolicy.org/article/global-dimensions-female-migration.

[17] Abdool Karim Q, Abdool Karim SS, Singh B, Short R, Ngxongo S. Seroprevalence of HIV infection in rural South Africa. AIDS. 1992;6(12):1535–9. . 1492937 10.1097/00002030-199212000-00018

[18] Kishamawe C, Vissers DC, Urassa M, Isingo R, Mwaluko G, Borsboom GJ, et al Mobility and HIV in Tanzanian couples: both mobile persons and their partners show increased risk. AIDS. 2006;20(4):601–8. doi: 10.1097/01.aids.0000210615.83330.b2 . 16470125 10.1097/01.aids.0000210615.83330.b2

[19] Lydie N, Robinson NJ, Ferry B, Akam E, De Loenzien M, Abega S. Mobility, sexual behavior, and HIV infection in an urban population in Cameroon. Journal of acquired immune deficiency syndromes (1999). 2004;35(1):67–74. Epub 2004/01/07. . 14707795 10.1097/00126334-200401010-00010

[20] Pison G, Le Guenno B, Lagarde E, Enel C, Seck C. Seasonal migration: a risk factor for HIV infection in rural Senegal. Journal of acquired immune deficiency syndromes (1999). 1993;6(2):196–200. Epub 1993/02/01. . 8433284

[21] Zuma K, Gouws E, Williams B, Lurie M. Risk factors for HIV infection among women in Carletonville, South Africa: migration, demography and sexually transmitted diseases. International journal of STD & AIDS. 2003;14(12):814–7. Epub 2003/12/18. doi: 10.1258/095646203322556147 . 14678589 10.1258/095646203322556147

[22] Khan MR, Patnaik P, Brown L, Nagot N, Salouka S, Weir SS. Mobility and HIV-related sexual behavior in Burkina Faso. AIDS and behavior. 2008;12(2):202–12. Epub 2007/10/31. doi: 10.1007/s10461-007-9314-8 . 17968650 10.1007/s10461-007-9314-8

[23] Bwayo J, Plummer F, Omari M, Mutere A, Moses S, Ndinya-Achola J, et al Human immunodeficiency virus infection in long-distance truck drivers in east Africa. Archives of internal medicine. 1994;154(12):1391–6. Epub 1994/06/27. . 8002691

[24] Hope KR Sr. Mobile workers and HIV / AIDS in Botswana. AIDS analysis Africa. 2000;10(4):6–7. Epub 2002/09/28. . 12349439

[25] Nunn AJ, Wagner HU, Kamali A, Kengeya-Kayondo JF, Mulder DW. Migration and HIV-1 seroprevalence in a rural Ugandan population. AIDS (London, England). 1995;9(5):503–6. Epub 1995/05/01. . 7639976

[26] Lurie MN, Williams BG, Zuma K, Mkaya-Mwamburi D, Garnett GP, Sweat MD, et al Who infects whom? HIV-1 concordance and discordance among migrant and non-migrant couples in South Africa. AIDS (London, England). 2003;17(15):2245–52. Epub 2003/10/03. doi: 10.1097/01.aids.0000088197.77946.ba . 14523282 10.1097/00002030-200310170-00013

[27] Camlin CS, Snow RC, Hosegood V. Gendered Patterns of Migration in Rural South Africa. Population, space and place. 2014;20(6):528–51. Epub 2014/10/22. doi: 10.1002/psp.1794 ; PubMed Central PMCID: PMCPMC4201383. 25332690 10.1002/psp.1794 PMC4201383

[28] Westercamp M, Jaoko W, Mehta S, Abuor P, Siambe P, Bailey RC. Changes in Male Circumcision Prevalence and Risk Compensation in the Kisumu, Kenya Population 2008–2013. Journal of acquired immune deficiency syndromes (1999). 2016 Epub 2016/09/16. doi: 10.1097/qai.0000000000001180 . 27632232 10.1097/QAI.0000000000001180 PMC5233580

[29] Programme” KNASC. Kenya AIDS Indicator Survey 2012. Nairobi: 2012.

[30] Kimanga DO, Ogola S, Umuro M, Kimondo L, Murithi P, Muttunga J, et al Prevalence and incidence of HIV infection, trends, and risk factors among persons aged 15–64 years in Kenya: results from a nationally representative study. JAIDS. 2014;66(Suppl: S13–S26). 24445338 10.1097/QAI.0000000000000124 PMC4794992

[31] Camlin C, El Ayadi A, Kwena Z, McFarland W, Johnson M, Neilands T, et al High Mobility and HIV Prevalence among Female Market Traders in East Africa in 2014. J Acquir Defic Syndr. 2017 4 15; 74 (5): e121–e128. PMC5340599. 10.1097/QAI.0000000000001252 PMC5340599 27875361

[32] Camlin CS, Kwena ZA, Dworkin SL, Cohen CR, Bukusi EA. "She mixes her business": HIV transmission and acquisition risks among female migrants in western Kenya. Social science & medicine (1982). 2014;102:146–56. Epub 2014/02/26. doi: 10.1016/j.socscimed.2013.11.004 ; PubMed Central PMCID: PMCPMC3935174. 24565152 10.1016/j.socscimed.2013.11.004 PMC3935174

[33] Camlin CS, Kwena ZA, Dworkin SL. Jaboya vs. jakambi: Status, negotiation, and HIV risks among female migrants in the "sex for fish" economy in Nyanza Province, Kenya. AIDS education and prevention: official publication of the International Society for AIDS Education. 2013;25(3):216–31. Epub 2013/05/02. doi: 10.1521/aeap.2013.25.3.216 ; PubMed Central PMCID: PMCPMC3717412. 23631716 10.1521/aeap.2013.25.3.216 PMC3717412

[34] Kulzer L, Penner JA, Marima R, Oyaro P, Oyanga AO, Shade SB, et. al Family model of HIV care and treatment: a retrospective study in Kenya. Journal of the International AIDS Society. 2012;15(1):8 Epub 2012/02/23. doi: 10.1186/1758-2652-15-8 ; PubMed Central PMCID: PMCPMC3298805. 22353553 10.1186/1758-2652-15-8 PMC3298805

[35] Hsieh FY, Bloch DA, Larsen MD. A simple method of sample size calculation for linear and logistic regression. Statistics in medicine. 1998;17(14):1623–34. Epub 1998/08/12. . 9699234 10.1002/(sici)1097-0258(19980730)17:14<1623::aid-sim871>3.0.co;2-s

[36] Fleiss J LBaPM. Statistical Methods for Rates and Proportions. 3rd Edition ed. New York:: John Wiley and Sons; 2003.

[37] Pison G, Le Guenno B, Lagarde E, Enel C, Seck C. Seasonal migration: a risk factor for HIV infection in rural Senegal. Journal of Acquired Immune Deficiency Syndromes. 1993;6(2):196–200. . 8433284

[38] Brockerhoff M, Biddlecom AE. Migration, Sexual Behavior and the Risk of HIV in Kenya.(Statistical Data Included). International Migration Review. 1999;33(4):833–56.

[39] Zuma K, Gouws E, Williams B, Lurie M. Risk factors for HIV infection among women in Carletonville, South Africa: migration, demography and sexually transmitted diseases. International Journal of STD & AIDS. 2003;14(12):814–7. doi: 10.1258/095646203322556147 . 14678589 10.1258/095646203322556147

[40] Boerma JT, Urassa M, Nnko S, Ng'weshemi J, Isingo R, Zaba B, et al Sociodemographic context of the AIDS epidemic in a rural area in Tanzania with a focus on people's mobility and marriage. Sexually Transmitted Infections. 2002;78 Suppl 1:i97–105. doi: 10.1136/sti.78.suppl_1.i97 . 12083453 10.1136/sti.78.suppl_1.i97 PMC1765836

[41] Lydie N, Robinson NJ, Ferry B, Akam E, De Loenzien M, Abega S. Mobility, sexual behavior, and HIV infection in an urban population in Cameroon. Journal of Acquired Immune Deficiency Syndromes. 2004;35(1):67–74. . 14707795 10.1097/00126334-200401010-00010

[42] Cohen CR, Montandon M, Carrico AW, Shiboski S, Bostrom A, Obure A, et al Association of attitudes and beliefs towards antiretroviral therapy with HIV-seroprevalence in the general population of Kisumu, Kenya. PloS one. 2009;4(3):e4573 Epub 2009/03/05. doi: 10.1371/journal.pone.0004573 ; PubMed Central PMCID: PMCPMC2649531. 19259267 10.1371/journal.pone.0004573 PMC2649531

[43] Cassels S, Jenness SM, and Khanna AS. Conceptual Framework and Research Methods for Migration and HIV Transmission Dynamics. AIDS and Behavior. 2014;18(12). 10.1007/s10461-013-0665-z PMC4029933 24257897

[44] Taylor JaB M. Towards comparative measures of circulation: Insights from Indigenous Australia. Population, space and place. 2012;18(5).

[45] Kwena ZA, Mwanzo IJ, Bukusi EA, Achiro LF, Shisanya CA. A cross-sectional survey of prevalence and correlates of couple sexual concurrency among married couples in fishing communities along Lake Victoria in Kisumu, Kenya. Sexually transmitted infections. 2014;90(2):139–44. Epub 2013/10/25. doi: 10.1136/sextrans-2013-051168 . 24154655 10.1136/sextrans-2013-051168 PMC5608652

